# Controlled-release gel encapsulation: an emerging technology for delivering beneficial microorganisms in sustainable plant disease management

**DOI:** 10.1007/s44297-026-00088-1

**Published:** 2026-09-22

**Authors:** Jingjing An, Xigang Wang, Gang Qiao, Chengjin Guo, Xiaojiao Li, Xianchao Sun

**Affiliations:** 1 Institute of Plant Protection, Ningxia Academy of Agriculture and Forestry Sciences, Yinchuan, Ningxia China; 2 Ningxia Key Laboratory of Plant Disease and Pest Control, Yinchuan, Ningxia China; 3 College of Plant Protection, Southwest University, Chongqing, China; 4 Key Laboratory of Agricultural Biosafety and Green Production of the Upper Yangtze River, Ministry of Education, Chongqing, China; 5 School of Biotechnology and Bioengineering, West Yunnan University, Lincang, China

**Keywords:** Controlled-release gel, Encapsulation technology, Plant diseases, Biological control

## Abstract

As climate change intensifies, the accelerated spread of plant diseases poses severe threats to global crop production, leading to substantial losses in yield, quality, and economic returns, thereby jeopardizing food security and agricultural sustainability. With increasing awareness of the environmental risks associated with chemical pesticides, eco-friendly alternatives are urgently needed. Although biological control represents a promising green strategy, its practical efficacy is often limited by poor microbial survival, low environmental stability, and short functional duration under field conditions. In response, controlled-release gel encapsulation technology has emerged as an innovative approach, integrating advances in materials science, microbiology, and plant pathology. By combining physical protection, controlled delivery, and induced resistance mechanisms, this technology significantly improves the viability, persistence, and performance of beneficial microorganisms in agricultural settings. This review systematically examines the application of controlled-release gel-based encapsulation of beneficial microbes in plant disease management, covering their mechanisms of action, material selection, encapsulation techniques, and practical benefits, while also addressing current challenges. Future perspectives should focus on the development of intelligent-responsive materials and the enhancement of the stability of target microorganisms. Moreover, the environmental safety and ecological sustainability of these materials should be evaluated using multi-omics and artificial intelligence technologies combined with systematic field validation and full life-cycle assessment. This review aims to establish a scientific foundation and offer strategic guidance for the practical application and optimization of controlled-release gel encapsulation technology in sustainable, green plant disease management.

## Introduction

### The threat of intensifying plant diseases to global crop production

The escalating incidence of plant diseases represents a critical threat to global agricultural systems, leading to severe deterioration in crop yield, product quality, and economic viability. These losses not only undermine food security but also exert profound pressures on ecological stability and the sustainability of agro-economic systems [1]. Globally, plant pests and diseases account for an estimated 20–40% of annual crop yield losses [2]. Notable examples include rice blast (*Magnaporthe oryzae*), which can reduce grain production by up to 30% [3], and wheat stripe rust (*Puccinia striiformis*), a recurrent epidemic across North Africa and South Asia that causes millions of tons of yield shortfalls each year [4]. Warming and associated climatic shifts, such as rising temperatures and altered precipitation patterns, accelerate the spread and virulence of plant diseases. In this context, effective management of plant diseases must be prioritized to safeguard the quality and health of agricultural products.

### Chemical pesticide dependency threatens agricultural sustainability

In contemporary agricultural practice, chemical pesticides remain the primary measure for pathogen control owing to their operational simplicity, cost-effectiveness, and broad-spectrum efficacy [1, 5]. Although these synthetic agents have substantially contributed to crop protection, their prolonged and often indiscriminate application has raised serious concerns regarding human health and ecosystem integrity [6, 7]. Extensive use of chemical pesticides not only contaminates soil and water but also drives the emergence of resistant pathogen strains [6–8], thereby undermining the long-term sustainability of chemical-based disease management [9, 10]. Furthermore, the accumulation of synthetic residues in the environment and within organisms can cause irreversible damage to ecosystems, leading to biodiversity loss [11, 12]. Moreover, the energy consumed during pesticide production and application, unintended harm to non-target organisms [13], and the production of mycotoxins and other virulence factors by many pathogenic fungi collectively pose severe risks to environmental safety and public health [14, 15]. As awareness of these hazards increases, so does the demand for non-chemical alternatives. In this context, biological agents represent superior options to synthetic pesticides, offering high efficacy, low residue levels, minimal toxicity to non-target organisms and humans, and low environmental impact [16].

### Constraints and pathways for advancing biological control applications

Biological control represents a promising plant protection strategy that enhances crop immunity via beneficial microorganisms or their metabolites, offering notable advantages such as high specificity and environmental sustainability [17, 18]. However, despite their proven efficacy under controlled conditions, biocontrol agents frequently face substantial hurdles in field applications, including poor environmental resilience, low post-introduction survival, and limited persistence of bioactive compounds–all factors that undermine their performance in real–world agricultural settings. A key factor underlying this instability is the inadequate colonization of plant roots by introduced microbial agents [7, 19]. Moreover, the efficacy of biocontrol is highly dependent on precise spatial and temporal delivery, as secondary metabolites are typically produced in low quantities and exhibit limited mobility in the soil environment [20]. Further complicating field deployment, environmental variables such as temperature and humidity fluctuations, variations in soil pH and microbial community structure, and plant-specific immune responses-pose major constraints that affect the consistency and reliability of biocontrol outcomes [21, 22].

Wettable powder formulations are among the most widely used commercial biocontrol agents against biotic stresses in agriculture. However, the production of granular formulations faces critical limitations. During processing, bioactive metabolites from biocontrol bacteria are susceptible to volatilization and degradation, but their poor aqueous solubility further compromises bioavailability [23, 24]. Moreover, in dry formulations, dehydration stress significantly decreases the viability of microbial cells, particularly non-spore-forming bacteria, leading to decreases in product quality, storage stability, and biological compatibility [25, 26]. Beyond these production challenges, field performance is constrained by a short residence time on target pests or pathogens, inefficient delivery of bioactive compounds to specific plant tissues, and limited resilience to environmental conditions such as UV radiation, temperature fluctuations, and rainfall, collectively compromising field efficacy [19, 27].

In this context, developing environmentally compatible, target-specific, and sustainable plant protection strategies has become imperative for advancing green agriculture [28]. Inspired by controlled-release principles from the pharmaceutical sciences, where dosage and release kinetics are designed to sustain therapeutic efficacy, similar approaches can be applied to optimize the delivery of biocontrol agents in plant disease management [29]. Currently, microencapsulation and targeted controlled-release systems for beneficial microorganisms have attracted considerable research interest and are being increasingly deployed in agricultural applications [30]. This innovative approach is emerging as a pivotal strategy for reconciling plant disease management with sustainable agricultural goals. As such, it offers a technological cornerstone for advancing eco-compatible crop protection and promoting sustainable food production systems [31, 32].

**Fig. 1:**
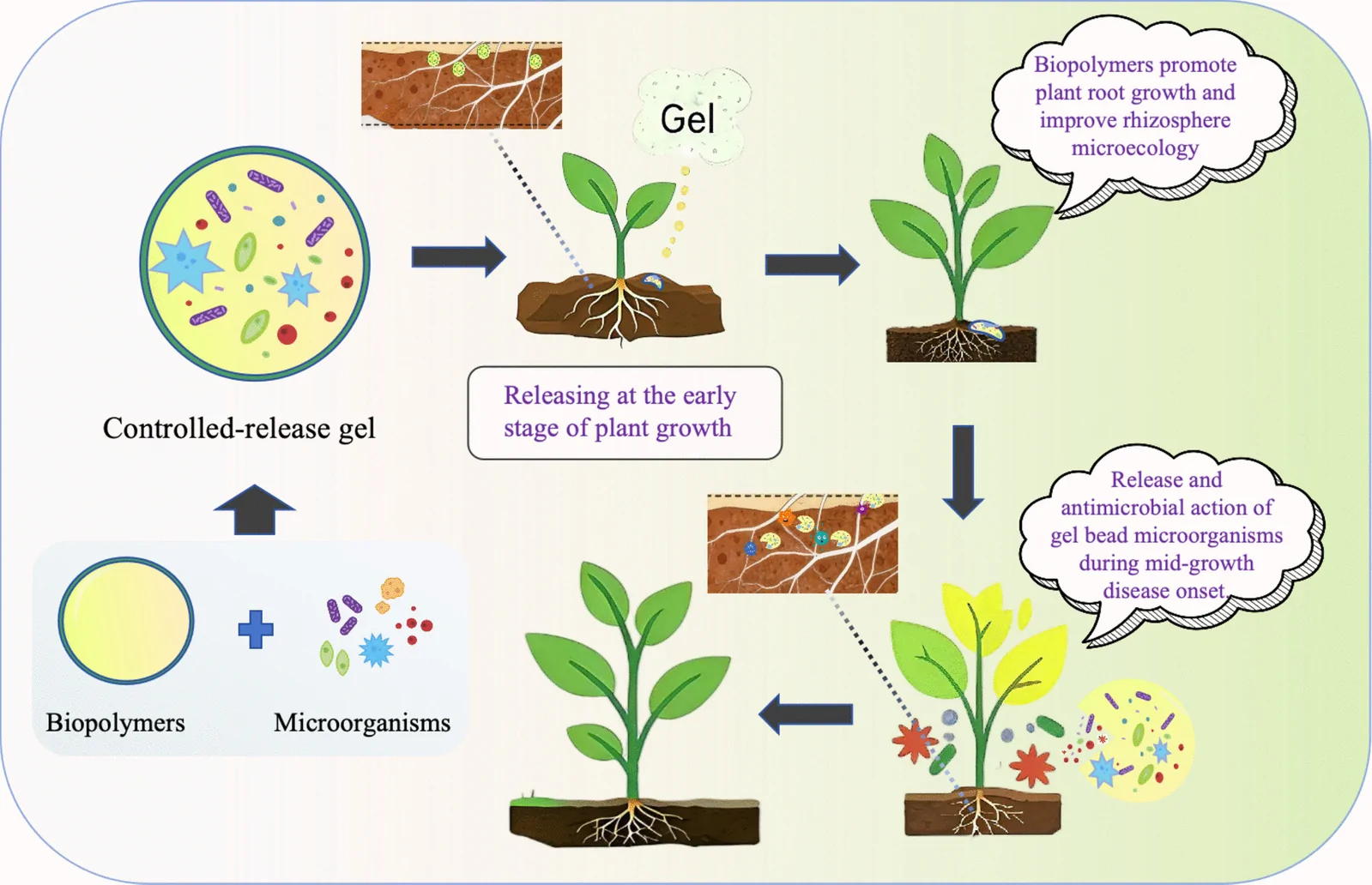
Schematic of controlled-release gel-encapsulated microorganisms in plant disease control. The diagram shows the fabrication process and functional mechanism of biopolymer-based microcapsules encapsulating beneficial microorganisms. The gel matrix (typically composed of alginate, chitosan, starch, or their composites) forms a three-dimensional cross-linked network that physically entraps microbial cells (e.g., *Bacillus, Pseudomonas, and Trichoderma*). Upon application to soil or plant surfaces, the gel gradually degrades or swells in response to environmental stimuli (such as moisture, pH, or microbial enzymes), enabling sustained and controlled release of the microorganisms. The released biocontrol agents then colonize the rhizosphere or phyllosphere; suppress pathogens through multiple mechanisms (including antagonism, competition, and induced resistance); and ultimately reduce disease incidence

At present, this technology is widely used in the medical field; however, its agricultural application remains nascent. This review focuses specifically on controlled-release gel encapsulation systems, which consist of a three-dimensional, water-swollen polymeric network (hydrogel) that entraps microorganisms. We believe that this review will accelerate the development of sustained-release gels for plant disease management and provide insights that may inspire multidisciplinary approaches in future biocontrol research [33, 34](Fig. 1).

## Controlled-release gel encapsulation of beneficial microorganisms for plant disease management

Extensive research has shown that compared with free bacterial cells, the ability to encapsulate biocontrol bacteria in biopolymers often results in superior efficacy, occasionally rivaling the performance of conventional chemical pesticides [35, 36]. Moreover, this approach significantly prolongs the shelf life and has demonstrated promising efficacy against plant diseases. For instance, spores of *Bacillus amyloliquefaciens* FZB42 encapsulated with carboxymethyl cellulose and alginate—polysaccharides derived from microbial membranes—exhibited strong inhibitory activity against alkaliphilic fungal pathogens, such as *Fusarium graminearum,* in vitro [37].

Compared with its free-cell counterparts, the microencapsulation of *Bacillus megaterium* in calcium alginate significantly improved bacterial survival under UV radiation and high-temperature stress. Under greenhouse conditions, the encapsulated formulation displayed efficacy against rice blast (*Magnaporthe oryzae*) comparable to that of conventional chemical fungicides [38]. Similarly, the encapsulation of *Bacillus thuringiensis* and *Bacillus subtilis* VRU1 within a starch–bentonite–alginate composite not only increased their tolerance to biotic and abiotic stresses but also increased their antagonistic activity against potato late blight (*Phytophthora infestans*) [39]. Compared with its non-encapsulated form, an encapsulated formulation of a *fluorescent Pseudomonas* strain also exhibited significantly enhanced biocontrol efficacy against *Fusarium graminearum* under greenhouse conditions, along with a prolonged shelf life [40]. Chitosan demonstrated broad-spectrum antimicrobial activity, effectively suppressing the growth of several phytopathogenic bacteria, including *Xanthomonas* spp. [41], *Pseudomonas syringae* [42], *Agrobacterium tumefaciens*, and *Erwinia carotovora* [43].

Nevertheless, it is important to recognize that the majority of these studies were conducted under controlled laboratory or greenhouse conditions, where environmental variables—such as temperature, humidity, UV radiation, and soil biotic interactions—are carefully regulated. Despite the encouraging results obtained under controlled settings, the scarcity of multi-season, multi-location field trial data is a notable gap in the current literature, as these conditions do not adequately reflect the complexity and variability of real-world agricultural fields.

## Mechanism

**Fig. 2:**
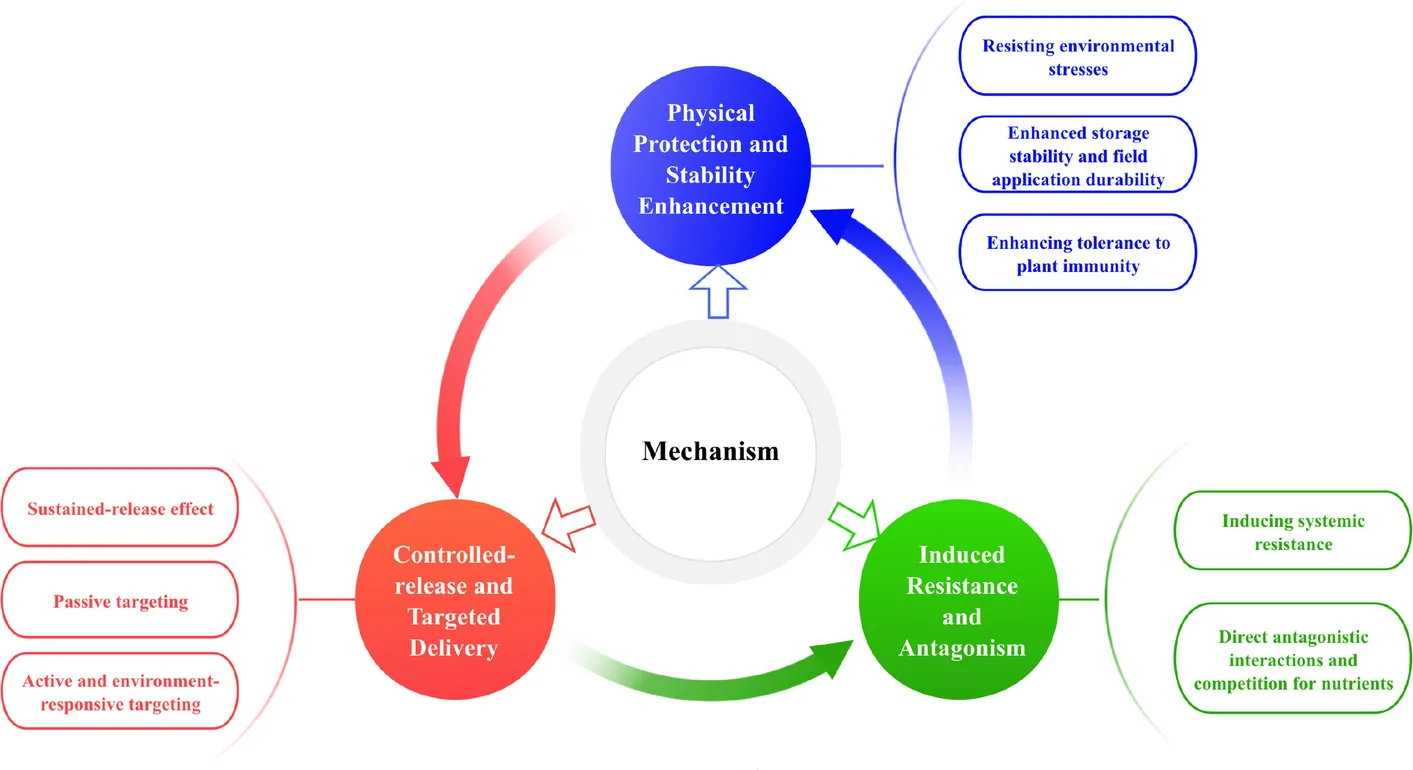
Mechanism of controlled-release encapsulation against plant diseases

Controlled-release gel encapsulation serves as a protective matrix for biocontrol formulations. Its ability to suppress plant diseases is significantly enhanced through synergistic interactions among multiple mechanisms, which primarily constitute the following aspects (Fig. 2).

### Physical protection against environmental stressors

A principal advantage of microencapsulation lies in its ability to establish a shielded microenvironment for microorganisms [44]. Robust electrostatic attraction between anionic polymers (e.g., alginate) and cationic polymers (e.g., chitosan) facilitates the formation of a denser capsule wall, substantially reinforcing both the protection of encapsulated microorganisms and the structural integrity of the gel matrix [45]. Consequently, the survival and persistence of microorganisms markedly improve throughout processing, storage, and field application [46, 47]. This protective effect manifests across two dimensions: spatial and temporal.

#### Spatial protection

Biopolymer encapsulation creates a physical barrier that effectively protects internal microorganisms from adverse environmental conditions, including UV radiation, extreme temperatures, humidity fluctuations, and pH changes [48, 49]. Studies have demonstrated that compared with free cells, *Bacillus megaterium* encapsulated in calcium alginate survives significantly better under UV and high-temperature stress [38]. Adding functional additives (bentonite, starch, or xanthan gum) to alginate or applying chitosan/starch–pectin coatings significantly improves the acid, UV, and thermal resistance of bacterial gels. Such modifications also increase rhizosphere colonization [50].

#### Temporal protection

Encapsulation technology temporarily isolates microorganisms from environmental stresses [51], markedly increasing survival rates and preserving biological activity under field conditions, thereby prolonging their functional duration and improving biocontrol efficacy [52, 53]. For instance, Perez et al. [54] demonstrated that the encapsulation of *Azospirillum brasilense* and *Pseudomonas fluorescens* within a chitosan–starch polymer matrix cross-linked with sodium tripolyphosphate could maintain bacterial viability for at least 12 months. In another study, Zou et al. [55] employed composite coatings such as starch/pectin or chitosan/poly-L-lysine to reinforce alginate-based encapsulation of *Bifidobacterium bifidum* F-35, significantly improving bacterial survival.

### Controlled-release and targeted delivery

The core advantage of encapsulation technology lies in enabling the controlled release of microorganisms at the right time, in the right place, and at a controllable rate. This is achieved primarily through the following mechanisms.

#### Sustained-release effect

The gel network formed by biopolymers (e.g., alginate and starch) delays the release of microorganisms, allowing sustained activity on the soil or plant surface over an extended period and thereby prolonging the duration of disease control. Studies confirm that microencapsulation significantly extends the release kinetics of beneficial bacteria, leading to enhanced and prolonged biocontrol efficacy against bacterial blight [56].

#### Passive targeting

The encapsulation system is preferentially absorbed via stomata, cuticular fissures, or roots, and may be systemically translocated, enhancing accumulation at infection sites [57].

#### Active and environment-responsive targeting

Surface functionalization of encapsulation carriers (e.g., chitosan) enhances binding affinity to specific pathogens or plant tissues, enabling site-specific accumulation. The development of intelligent encapsulation systems can respond to stimuli within the disease microenvironment (e.g., pH variations or pathogen-secreted specific enzymes). This allows on-demand release of microorganisms at infection sites [58].

### Induced resistance and antagonism

Encapsulation materials possess inherent biological activity and synergize with encapsulated microorganisms to enhance plant disease control efficacy.

#### Inducing systemic resistance

Numerous biopolymers, including chitosan, intrinsically function as potent “resistance elicitors”. They activate plant innate immune and defense systems through a cascade of biochemical and molecular signaling events. These biopolymers promote the synthesis and accumulation of phytoalexins in various host tissues and further elicit key defense responses, including the triggering of callose deposition, lignification, and the production of protease inhibitors [59]. Sathiyabama et al. [60]. demonstrated the potential of chitosan as an effective exogenous elicitor for enhancing the production of curcumin in turmeric. Notably, the immune-inducing function of chitosan as a biopolymer and its role as a physical barrier for encapsulated microorganisms are not contradictory. Instead, they operate at different levels and serve distinct purposes [60, 61].

#### Direct antagonistic interactions and competition for nutrients

The application of encapsulation materials, while effectively enhancing soil structure, also induces alterations in the rhizosphere environment and microbial equilibrium. This selectively favors beneficial microbes while suppressing pathogenic microbes. Evidence shows that the population of *Bacillus subtilis* in strawberry-cultivated soil increases following the incorporation of chitosan (CHI) [62]. Moreover, owing to the chelating properties of biopolymers, CHI can mitigate the phytotoxicity caused by toxic elements, fertilizers, and pesticides.

## Encapsulation materials and encapsulation methods

### Encapsulation materials

**Fig. 3:**
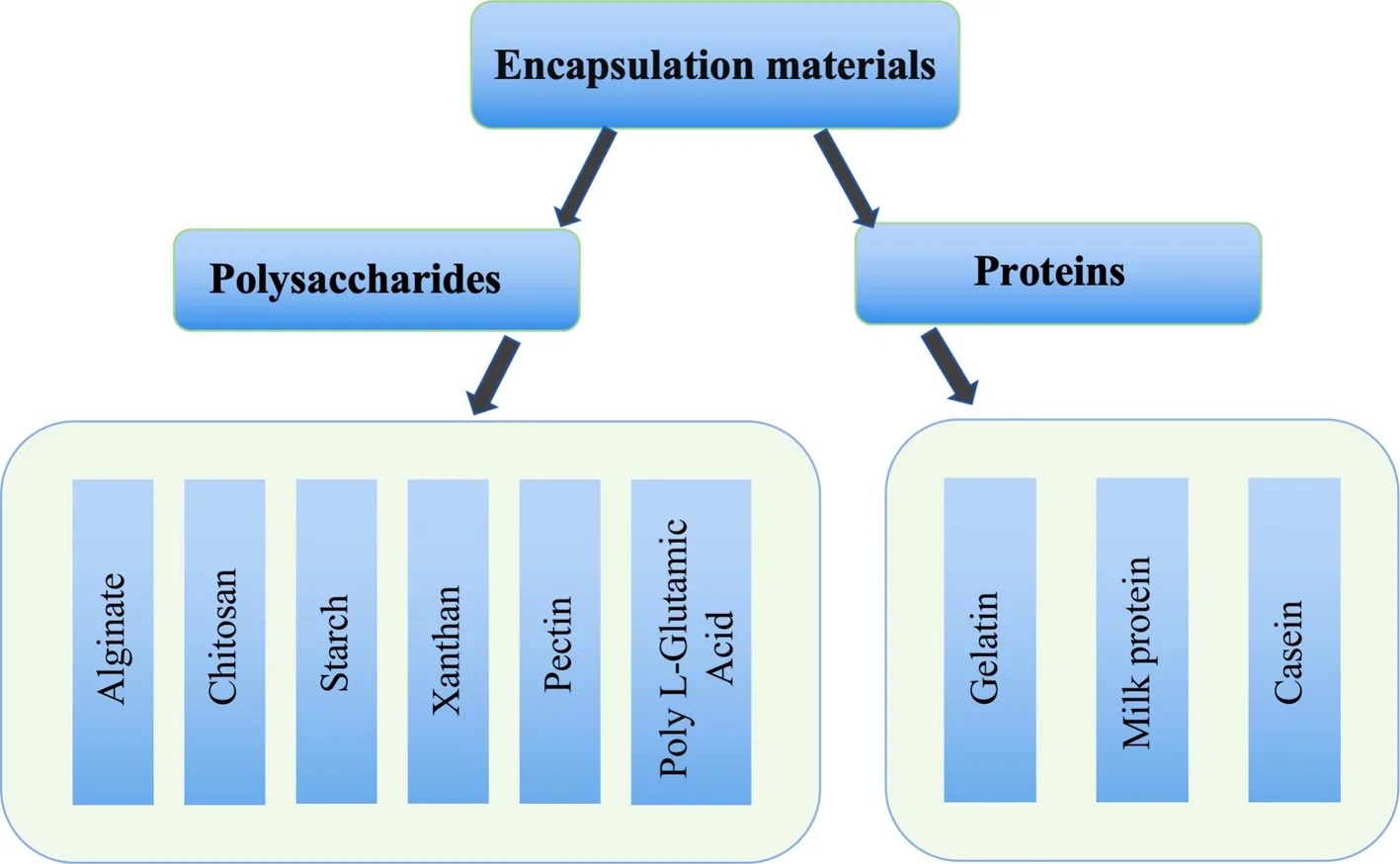
Biopolymers as controlled-release encapsulation materials

The purpose of encapsulation is to establish a protective microenvironment that sustains the viability of beneficial microorganisms throughout processing and storage while enabling their targeted release at the desired site [63]. To ensure the viability, functionality, and efficacy of encapsulated microorganisms in specific application contexts, the encapsulation materials (Fig. 3) must exhibit a set of critical attributes. These include biocompatibility, biodegradability, mechanical stability, protective efficacy toward microorganisms, controlled release kinetics, economic feasibility and ready availability [27, 64].

#### Starch

Among the various biopolymers employed for encapsulation, starch stands out as a highly promising carrier because of its abundant availability, cost-effectiveness, and excellent biocompatibility [65]. It is widely distributed in plant organs, including fruits, tubers, roots, leaves, and seeds. As a major agricultural commodity, large-scale production of starch is economically feasible. It is edible, non-toxic, hypoallergenic, and fully biodegradable. Consequently, starch-based microbial formulations are environmentally friendly and leave no chemical pesticide residues, meeting the requirements of agricultural product safety and sustainable farming. Starch also has favorable film-forming and gelling properties. These processes enable the formation of stable microcapsules or hydrogel network structures through various techniques—such as extrusion and emulsion cross-linking—to efficiently encapsulate bioactive ingredients [66].

Despite the numerous advantages of starch as an encapsulation material, its limited mechanical strength and poor water resistance restrict its application performance and stability under complex environmental conditions. To overcome these challenges, physical or chemical modification strategies are typically adopted. Cross-linking with other polymers is among the most effective approaches. As demonstrated by Tan et al. [65, 67], cross-linking with poly(N-isopropylacrylamide) can increase the mechanical strength of starch-based hydrogels by up to 200-fold. Similar improvements have been observed when starch is combined with sodium alginate, bentonite, or chitosan. These combinations facilitate the formation of a more compact and stable encapsulation matrix, which enhances the protection of encapsulated microorganisms [48, 67].

#### Chitosan

Chitosan, a naturally derived polysaccharide produced through the alkaline deacetylation of chitin [59], exhibits distinctive biological and physicochemical characteristics that confer considerable potential for agricultural biotechnology, particularly in the encapsulation and delivery of biological control agents (BCAs) [68, 69]. As an encapsulation matrix, chitosan offers ideal attributes, including non-toxicity, biodegradability, biocompatibility, ease of processing, and relatively low cost. These attributes align with the criteria for environmentally sustainable agricultural inputs [70, 71]. Chitosan possesses abundant amino groups, which render it readily soluble and cationic under acidic to neutral conditions. This property facilitates the formation of stable ionic hydrogels via electrostatic interactions with anionic polymers, such as alginate and xanthan gum. Such versatile gel-forming properties have led to its wide application in the agriculture, food, and pharmaceutical sectors [72]. Beyond serving as a carrier, chitosan acts as an effective biostimulant. It systemically induces plant innate immunity and enhances resistance to pathogens [73, 74]. Chitosan also promotes plant growth and development, providing the dual benefits of resistance induction and growth enhancement [75].

In the study by Li et al. [41], *Lactobacillus casei* ATCC 393 was encapsulated in a matrix of sodium alginate, chitosan, and carboxymethyl chitosan. After 28 days of storage at 4 °C, cell viability remained high, reaching up to 10^8^ cfu/g. In a parallel investigation, *Methylobacterium oryzae* was encapsulated with varying concentrations (0.3%–3%) of chitosan combined with 2% alginate. Following 3 months of storage, the bacterial viability was maintained at 80%, and the treatment significantly promoted the growth of tomato seedlings [76].

#### Sodium alginate

Among known polymer carriers, alginate is among the most widely used materials for microbial encapsulation [77, 78]. Its primary advantage lies in its ability to undergo rapid ionic gelation through cross-linking with divalent cations, most commonly Ca^2+^—under mild conditions. This process results in the formation of a hydrogel network around microbial cells, enabling efficient encapsulation and immobilization without organic solvents or elevated temperatures, thus minimizing the impact on microbial viability [79]. Beyond encapsulation, alginate-based hydrogels are highly hydrophilic and possess a three-dimensional porous structure, making them effective soil moisture regulators [80].

Despite its numerous advantages, alginate has several inherent limitations in practical applications. Alginate hydrogels are unstable under alkaline conditions. Ca^2+^ interacts with OH^–^ to form insoluble precipitates, which compromises the integrity of the gel network [78]. This instability restricts their use in alkaline soils. Furthermore, the alginate gel matrix has a relatively loose and porous structure. Although this facilitates nutrient diffusion, it also weakens the barrier properties of microcapsules, leading to premature leakage of bioactive compounds and a reduced release duration [81]. Pure alginate gels also lack mechanical robustness, making them susceptible to rupture under physical agitation or abrasion by soil particles. Their relatively rapid degradation further limits their applicability in long-term storage or sustained release. Additionally, chelating agents (e.g., citrates) and competing ions (e.g., Mg^2+^) can sequester calcium ions from the gel network, disrupting the cross-linked matrix and affecting the integrity of the gel [82].

To address these limitations, alginate is often combined with other functional materials to develop advanced hybrid encapsulation systems [81]. For example, alternating multilayer coatings of alginate and chitosan markedly improved the survival of *Bifidobacterium* breve under simulated gastric conditions characterized by low pH [55]. Such coating designs effectively reduce gel porosity, thereby enhancing both the protection of encapsulated contents and their controlled release [72]. The physical blending or chemical cross-linking of alginate with more stable or functionally complementary biopolymers, such as gelatin, starch, lignin, or xanthan gum, enables the formation of interpenetrating polymer networks (IPNs). This strategy not only significantly improves the mechanical strength and structural stability of the gel but also compensates for the shortcomings of individual components. For instance, gelatin imparts enhanced elasticity, whereas chitosan increases matrix density and confers additional functional properties, including the induction of plant resistance [83].

#### Milk proteins

Owing to their favorable physicochemical properties, milk proteins serve as effective carrier materials for microbial immobilization. Their excellent gel-forming ability and high biocompatibility make them particularly suitable for entrapping and protecting sensitive strains. For instance, studies have successfully encapsulated bacterial strains such as *Lactobacillus rhamnosus* GG and *Bifidobacterium paracasei* F19 within milk protein-based systems [84, 85].

#### Other biopolymers

Pectin, a complex polysaccharide found in plant cell walls, consists primarily of galacturonic acid units. Owing to its biocompatibility, biodegradability, and outstanding gelling capacity, it has significant potential for microbial encapsulation [86, 87]. The gelation mechanism of pectin typically involves ionic cross-linking, which is typically mediated by Ca^2+^, or interactions with other biopolymers such as proteins [88, 89]. In practice, however, both the encapsulation efficiency of pectin and the viability of encapsulated microorganisms are affected by multiple factors. These include the type of pectin (e.g., degree of esterification), the nature and concentration of cross-linking agents, the encapsulation technique employed, and the intrinsic properties of the microorganisms [90]. Consequently, it is essential to investigate pectins from diverse sources and their chemical modifications to optimize their gelling behavior and protective performance. For instance, developing multilayer or composite encapsulation systems incorporating other biopolymers—such as chitosan, alginate, or proteins—could enhance protective efficacy and enable more precise controlled release [91].

Xanthan gum is an exopolysaccharide produced by *Xanthomonas campestris* through aerobic fermentation. Its main structural unit consists of two glucose molecules, two mannose molecules, and one glucuronic acid [92]. This unique structure confers pseudoplasticity, high viscosity, and good stability under high-salt conditions and over a wide range of pH values. The rigidity of its molecular chain and the double helix structure it forms in aqueous solutions provide excellent gel-forming ability and mechanical strength. These properties are crucial for microbial encapsulation. Combining xanthan gum with other biopolymers can produce synergistic effects, further optimizing system performance. For example, compared with the control, the use of *Bacillus subtilis* with xanthan gum and carboxymethyl cellulose significantly reduced the number of cucumber root-knot nematodes under greenhouse conditions [93].

Poly-L-glutamic acid (PGA), a naturally occurring polypeptide, is characterized by its non-toxicity, hydrophilicity, and structural composition of L-glutamic acid units linked by amide bonds [94]. This water-soluble, edible, and biodegradable polymer is biosynthesized by *Bacillus* species [95]*.* Owing to its pronounced biodegradability, PGA represents a promising material for encapsulation and tissue engineering applications [96, 97].

To systematically compare the differences in encapsulation performance among the aforementioned biopolymers, Table 1 summarizes key quantitative indicators, including encapsulation efficiency, particle size, and viability after storage for various material systems.

**Table 1:** Comparison of the physicochemical properties and microbial viability of different biopolymers encapsulation materials

Encapsulation material	Encapsulation efficiency (EE, %)	Particle size (μm)	Viability after storage (%)	Storage conditions	Mechanical strength	Controlled-release performance	Main limitations
Sodium alginate	93 ± 0.8	2940	77 ± 0.9 (30 days)	Room temperature	Low	Medium (fast release)	Unstable in alkaline conditions; high porosity
Sodium alginate + xanthan gum	95.13 ± 0.44	318.40 ± 10.30	—	Room temperature	Medium–high	Medium-slow	High viscosity; difficult processing
Sodium alginate + chitosan	70.06 ± 0.64	215.20 ± 1.87	—	Room temperature	Medium–high	Slow	Relatively low encapsulation efficiency
Sodium alginate + starch	93.80 ± 0.63	221.10 ± 3.89	91 ± 0.8 (30 days)	Room temperature	Medium	Medium-slow	Water resistance needs improvement
Sodium alginate + gum arabic	93.06 ± 0.74	188.20 ± 4.98	—	Room temperature	Medium	Medium	Higher cost
Sodium alginate + sodium caseinate	88.40 ± 0.63	1307.30 ± 126.70	—	Room temperature	Low	Fast	Excessive particle size
Sodium alginate + carrageenan	77.90 ± 1.04	224.30 ± 6.88	—	Room temperature	Medium–high	Slow	Low encapsulation efficiency
Sodium alginate + starch + chitosan	—	—	log 6.35 CFU/g (135 days)	Stored after freeze-drying	Medium–high	Medium-slow	Complex optimization of multi-component system
Chitosan + starch	—	—	> 12 months	Room temperature	Medium	Slow	Limited mechanical strength

The data are compiled from different studies with varying microbial strains, encapsulation conditions, and testing methods. “—” indicates that the value was not reported in the original study

As shown in Table 1, pure alginate offers high encapsulation efficiency but suffers from low mechanical strength, rapid release, and instability in alkaline environments. Coating alginate with chitosan significantly improved bacterial viability after storage and enhanced mechanical integrity. Blending alginate with xanthan gum or starch increases its encapsulation efficiency and slows its release but introduces drawbacks such as high viscosity or poor water resistance. Notably, chitosan-based nano-coatings achieve the highest efficiency and excellent thermotolerance but require complex fabrication. Starch–chitosan systems can sustain viability for more than 12 months but exhibit limited mechanical strength. Overall, composite matrices generally outperform single-component systems in terms of protection and controlled release, yet no universal formulation exists—material selection must balance encapsulation efficiency, mechanical robustness, release kinetics, and process scalability according to specific application needs.

### Gel encapsulation techniques

Encapsulation of functional materials can be achieved using a range of established techniques, including emulsification, freeze-drying, extrusion coating, spray chilling, fluidized bed coating, coacervation, spray drying, and thermogelation [98]. Among these methods, ionic gelation and extrusion are typically used to encapsulate beneficial microorganisms [99].

#### Ionic gelation

Ionogelation has emerged as a globally established classical method because of its operational simplicity, mild processing conditions, and cost-effectiveness [100]. The underlying mechanism involves physical cross-linking driven primarily by electrostatic interactions. Central to this process is the ionic bridging between multivalent cations—most commonly divalent ions such as Ca^2+^—and anionic sites along the biopolymer chains, notably the guluronic acid (G) residues in alginate [101]. As cations coordinate with these negatively charged functional groups, they facilitate the formation of a three-dimensional “egg-box” architecture between adjacent polymer strands, resulting in the rapid transition from a liquid solution to a solid hydrogel matrix. This cross-linking reaction is notably mild, avoiding high temperatures or organic solvents, and thus preserves the biological activity of encapsulated microorganisms [102].

#### Extrusion

Extrusion is a physical encapsulation technique for immobilizing living microbial cells. In this process, a polymeric solution—typically sodium alginate—containing viable microorganisms is extruded under controlled pressure through a syringe needle or peristaltic pump. This generates uniform droplets through a nozzle of defined diameter. The droplets are then introduced into a gelling bath with divalent cations, such as CaCl_2_ [103]. Upon contact with calcium ions, an instantaneous ionic cross-linking reaction occurs, triggering immediate gelation at the droplet interface, followed by progressive inward solidification. This results in structurally stable hydrogel beads. The entire procedure is conducted under ambient or mild thermal conditions, thereby preventing thermal degradation and minimizing cellular damage [50], thus maintaining high cell viability. However, the conventional dropwise extrusion technique suffers from low production throughput and constrained bead formation rates, making it insufficient for large-scale industrial applications [104].

### Related non-gel encapsulation methods

#### Emulsification

Emulsification technology is a key physicochemical encapsulation technique. It is widely employed to immobilize biocontrol agents, enhancing bacterial survival and stability during processing, storage, and application. The core principle involves dispersing a liquid phase containing bioactive substances (e.g., microbial cells in an aqueous phase) into an immiscible liquid phase (such as oil), thereby forming a homogeneous emulsion. The dispersed droplets are then solidified physically or chemically to produce water-insoluble microspheres or microcapsules [36]. Pour et al. [40]. revealed that strains of *Pseudomonas fluorescens* T17-4 and VUPF5 encapsulated within alginate–gelatin matrices via emulsification retained high stability after 6 months of storage at ambient temperature and exhibited markedly improved viability under greenhouse conditions.

#### Spray drying

Spray drying is a widely utilized dehydration technique that converts liquid substances into powders [105, 106]. Notable advantages of spray drying encompass rapid processing, high efficiency, reproducibility, operational simplicity, scalability, and low production costs [107, 108]. The primary limitations are the potential loss of bacterial viability due to elevated processing temperatures and the limited applicability to small-scale operations [109]. Studies indicate that combining lipid-based delivery systems with spray drying can enhance the stability, bioavailability, and targeted release of bioactive compounds [110]. For example, Costa et al. [111] demonstrated that spray-dried microencapsulation of *Enterobacter cloacae* strains resulted in high viability and post-rehydration survival rates.

Significant differences exist among the different encapsulation techniques in terms of particle size control, production capacity, cost, and impact on microbial viability [102]. Table 2 provides a systematic comparison of the advantages, disadvantages, and applicable scenarios of the major techniques.

**Table 2:** Comparison of advantages, disadvantages, and applicable scenarios of major encapsulation techniques

Encapsulation technique	Principle	Particle size range	Cell viability retention	Production capacity	Cost	Main advantages	Main disadvantages	Applicable scenarios
Extrusion	Physical extrusion + ionic cross-linking	2–5 mm	High (> 90%)	Low	Low	Simple operation, mild conditions, no organic solvents	Low throughput, large particle size, difficult to scale up	Laboratory research, small-scale production
Emulsion	Emulsification + solidification	< 300 μm	Medium–high	High	Medium	Scalable, controllable particle size, suitable for lipid systems	Emulsifiers may damage cells, significant liquid loss	Pilot and industrial production
Spray drying	Thermal atomisation and drying	< 100 μm	Medium–low (26–76%)	Very high	Low	Continuous operation, high efficiency, uniform particle size, easy storage	High temperature damages cells, not suitable for heat-sensitive strains	Large-scale industrial production
Freeze-drying	Low-temperature sublimation drying	Variable	High (> 93%)	Low	High	High cell viability retention, good durability	Time-consuming, high cost, ice crystals may damage cells	Long-term preservation of high-value strains
Layer-by-layer assembly	Electrostatic layer-by-layer adsorption	Variable	High (~ 95%)	Low	Medium–high	Precise controlled release, strong protection	Tedious, time-consuming	Functional precision encapsulation

A comparison of encapsulation techniques reveals clear trade-offs among throughput, cell viability, and scalability. Extrusion offers high viability and simplicity but suffers from low productivity, limiting it to laboratory-scale use. Emulsion and spray drying are scalable, with the emulsion providing medium–high viability and a controllable particle size, whereas spray drying achieves very high throughput at the cost of significant cell damage due to thermal stress. Freeze-drying preserves viability but is time-consuming and expensive and best suited for high-value strains. Layer-by-layer assembly enables precisely controlled release but is labor-intensive. In essence, no single method is suitable for all applications—selection should depend on the production scale and heat sensitivity of the target microorganism.

## Advantages and challenges

### Advantages

#### Interdisciplinary integration for addressing agricultural challenges

Controlled-release gel encapsulation technology is an advanced formulation for the biological control of plant diseases. It integrates cutting-edge knowledge from materials science, microbiology, plant pathology, and agricultural engineering. From a materials perspective, research focuses on developing novel biopolymers and smart responsive materials to increase the mechanical strength, controlled-release behavior, and environmental adaptability of carriers [88]. In microbiology, the focus lies in screening and evaluating highly active biocontrol strains from genera such as *Trichoderma*, *Bacillus*, and *Pseudomonas*, along with investigating their survival, colonization, and metabolic dynamics within encapsulation systems [38, 39, 42]. From the perspective of plant pathology, targeted delivery and stimulus-responsive release strategies are based on the infection cycles and pathogenic mechanisms of specific pathogens. Moreover, agricultural engineering principles are applied to optimize and integrate key encapsulation processes, such as extrusion molding, emulsification cross-linking, and spray drying. This bridges the gap between laboratory development and large-scale field application [98]. In summary, this technology provides a systematic solution that reflects the characteristics of interdisciplinary integration for sustainable plant disease management.

#### Multiple synergistic mechanisms for comprehensive plant disease management

The controlled-release gel encapsulation system is not simply a physical barrier but operates through a sophisticated multi-mechanism synergy [53]. The gel matrix shields encapsulated microorganisms from abiotic stresses, such as ultraviolet radiation, elevated temperatures, drought, and pH fluctuations. This protection substantially enhances microbial viability during storage and under field conditions [58, 112]. Through mechanisms such as controlled release, targeted delivery, and induced resistance, biocontrol agents are gradually liberated, precisely positioned, and able to systemically activate the plant’s innate immune responses, thus prolonging their duration of action. The encapsulation system modulates the rhizosphere microenvironment, promoting the colonization of beneficial agents and intensifying competition with pathogens for niches and nutrients [54, 59]. This suppresses disease occurrence at the source. In summary, by integrating these synergistic mechanisms, the system significantly improves the stability and persistence of biocontrol efficacy, offering a viable strategy to reduce reliance on chemical fertilizers and pesticides [83].

#### Biopolymer synergy: paving the way for eco-friendly materials innovation

Controlled-release gel encapsulation technology effectively addresses the inherent limitations of single-component materials—such as mechanical strength, inadequate environmental stability, and unsatisfactory controlled-release performance. This is achieved through the rational design and synergistic combination of biopolymers with complementary properties, including alginate, chitosan, and starch. This strategy has led to a range of advanced composite carriers that markedly improve microbial survival under stressful conditions and increase the colonization capacity of the rhizosphere. Innovatively, it introduces a novel concept of substituting conventional chemical materials with biopolymers [65, 113]. Not only does this approach provide a viable solution to the industry’s persistent challenge of poor field stability in biocontrol formulations, but it also marks a shift from chemical reliance toward an eco-compatible paradigm [114]. These findings offer theoretical support for the development of environmentally friendly biopesticides and align well with the principles of sustainable agriculture [115].

### Challenges

Controlled-release gel encapsulation technology has shown great potential for enhancing the efficacy of biocontrol formulations. Nevertheless, the practical implementation of this technology faces several barriers that must be overcome to bridge the gap between laboratory research and large-scale field applications.

#### Challenges in structural stability and loading capacity

Pure alginate gels have low inherent mechanical strength and high porosity. They are susceptible to structural collapse under soil mechanical stress and show significantly compromised stability under alkaline conditions [78, 81]. In contrast, pure starch gels are characterized by poor water resistance and rapid biodegradation. These properties undermine their capacity for long-term protection [65, 66]. With respect to loading capacity, the intrinsic physicochemical properties of these natural polymers limit their ability to efficiently encapsulate and immobilize microorganisms at high densities. This frequently results in low encapsulation efficiency and premature leakage of active agents [116]. Production methods also limit encapsulation performance. Gentle extrusion preserves microbial viability but is inefficient and restricts large-scale use.

#### Challenges in microbial stability under long-term encapsulation

The long-term encapsulation of microorganisms imposes multiple stresses on their viability. These stresses arise primarily from material degradation and microenvironmental imbalance. For instance, certain gel matrices may undergo gradual degradation, releasing byproducts that adversely affect microbial survival. Non-optimized alginate gels may release low-molecular-weight compounds under conditions such as high salinity or elevated temperature, which have been shown to inhibit microbial growth [77, 83]. On the other hand, prolonged encapsulation can deteriorate the internal physicochemical environment. As metabolic activities progress, microbial metabolites (e.g., organic acids) accumulate and cause localized acidification. This inhibits microbial function. In the case of aerobic microorganisms, oxygen diffusivity restricted through the gel network often creates a hypoxic or anoxic microenvironment. This severely impairs metabolic efficiency and reproductive capacity. Furthermore, nutrient depletion and the buildup of metabolic waste products constitute key intrinsic factors that reduce microbial activity over extended encapsulation periods [49, 100].

#### Challenges for the stability of efficacy in complex field environments

Encapsulation formulations that demonstrate efficacy under laboratory and greenhouse conditions may not translate effectively to complex and heterogeneous field environments. The core principle of controlled-release gel encapsulation is its ability to achieve “demand-based release” of active ingredients. However, release kinetics are strongly influenced by fluctuating field conditions, such as temperature, humidity, and soil pH. This makes precise control over release rates a significant challenge [99]. Existing controlled-release materials often lack the adaptability required to perform consistently across diverse crop and soil systems. Extreme temperatures, UV radiation, rainfall leaching, and microbial activity can accelerate the physical disintegration or chemical degradation of the encapsulation matrix. This substantially reduces the longevity of the controlled-release effect. For instance, water-soluble encapsulants may be rapidly dissolved upon rainfall, causing premature release of excessive amounts of active ingredients and compromising application efficiency [22, 36]. Therefore, the colonization efficiency and ecological persistence of encapsulated microbial agents across various crop–soil regimes necessitate comprehensive field validation.

#### Challenges for efficacy stability in complex field environments

Although controlled-release gel encapsulation technology offers a more environmentally benign alternative to conventional pesticide or fertilizer application, its long-term effects on soil microbial communities and non-target organisms remain insufficiently elucidated. As foreshadowed in Sect. 2.1, the encouraging efficacy data obtained under laboratory and greenhouse conditions often fail to translate into equivalent performance in real agricultural fields. This lab-to-field translational gap represents perhaps the most formidable barrier to the practical implementation of controlled-release gel encapsulation technology. Encapsulation formulations that demonstrate robust disease suppression under controlled settings may prove inconsistent or ineffective when confronted with the complexity and heterogeneity of field environments [99]. During environmental degradation, certain synthetic or modified polymers may release by-products such as microplastics, oligomers, or chemical monomers. The environmental behavior of these substances—including their migration, transformation, and bioaccumulation potential in soil—has not been comprehensively assessed, raising concerns over the risks to soil fauna (e.g., earthworms), arthropods, and groundwater systems [9, 17]. The transfer and amplification of encapsulation materials and active ingredients in complex food webs, particularly under chronic low-dose exposure scenarios, require systematic evaluation through standardized field trials [22, 99].

## Conclusions and future perspectives

Controlled-release gel encapsulation technology integrates principles from materials science, microbiology, and plant pathology. This provides an innovative and sustainable strategy for eco-friendly management; by combining physical protection, controlled release, and induced resistance, this technology markedly improves the stability, viability, and field efficacy of biocontrol agents. Nevertheless, several challenges remain for practical implementation. At the material level, the structural integrity and loading efficiency of the gel matrix require further improvement. At the microbial level, challenges such as microenvironmental imbalance and the long-term maintenance of viability under encapsulation have yet to be fully resolved. In field applications, the effects of complex environmental factors on release kinetics and control performance require more systematic investigation. A deeper understanding of the environmental behavior and long-term ecological impacts of encapsulation materials is essential. In the future, interdisciplinary innovation will be crucial for establishing controlled-release gel encapsulation technology as a core component of chemical pesticide reduction and replacement strategies, thereby supporting the safe production of food and promoting sustainable agricultural development.

### Optimization and innovation of the encapsulation materials and processes

As discussed in Sect. "Challenges in structural stability and loading capacity", the practical application of gel encapsulation is constrained by material-related limitations. To address these bottlenecks, future research should prioritize the development of advanced composite gel systems through the synergistic combination of complementary biopolymers. For example, incorporating functional additives such as bentonite, lignin, or cellulose nanocrystals into alginate or starch matrices can significantly increase mechanical robustness and structural stability. Multilayer coating strategies—such as the use of alternating layers of chitosan‒alginate—can effectively reduce gel porosity and improve the protective capacity for encapsulated microorganisms [117]. Additionally, the exploration of novel cross-linking chemistries, including dynamic covalent bonds or metal‒ligand coordination, may offer new avenues for constructing gels with tunable mechanical properties and self-healing capabilities.

From a processing perspective, a persistent challenge is reconciling the low throughput of mild techniques (which preserve high microbial viability) with the high-efficiency but microbial activity-damaging conditions of scalable methods [118]. Future efforts should focus on developing hybrid or multistage processing strategies that can achieve both high production efficiency and satisfactory cell survival. The systematic screening of biocontrol strains, particularly spore-forming bacteria such as *Bacillus* spp. and fungi such as *Trichoderma* spp., should be integrated with material selection to establish optimal strain–matrix combinations [119, 120]. Such matching strategies provide a theoretical foundation for the rational design of encapsulation systems and collectively increase their performance and application potential.

### Intelligent responsive systems for maintaining target microorganism stability

A major barrier to field efficacy, as identified in Sect. "Challenges for the stability of efficacy in complex field environments", is the inability of current encapsulation systems to achieve precise, environment-triggered release under fluctuating field conditions. The ideal gel system should release its microbial payload at the “right time, in the right place, and at the right dose.” Future work should focus on designing matrices that respond to relevant stimuli, such as pathogen-secreted lytic enzymes (e.g., cellulases or pectinases), pH shifts induced by root exudates during infection, or local temperature and humidity changes. Gels cross-linked with enzyme-labile moieties can undergo controlled degradation upon contact with pathogen-secreted enzymes, enabling targeted release at infection sites [121]. pH-responsive polymers can be engineered to release microorganisms under mildly acidic conditions that are often associated with root pathogen activity. Such “on-demand” release mechanisms would not only enhance control efficacy but also minimize wasteful dispersal and reduce application frequency.

To improve long-term microbial stability, research should also focus on constructing multi-phase microenvironments within the gel matrix. This can be achieved, for example, by incorporating protective agents (e.g., trehalose or skim milk) or co-encapsulating synergistic microbial consortia [122, 123]. These strategies could maintain a favorable physicochemical microenvironment, mitigating acidification, oxygen depletion, and nutrient limitation during prolonged storage (as noted in Sect. "Challenges in microbial stability under long-term encapsulation"). Through the rational design of smart responsive systems, a shift from “continuous release” to “on-demand release” can be realized. This offers a promising pathway for sustaining long-term microbial functionality [124].

### Systematic assessment of long-term field performance and ecological safety

To facilitate the successful translation of controlled-release gel encapsulation from laboratory research to agricultural practice, it is essential to establish a comprehensive evaluation framework that addresses both long-term field efficacy and environmental safety.

Long-term field validation should be prioritized in future research. Systematic, multi-season, multi-location field trials should be conducted across diverse soil types, climate conditions, and cropping systems. Experimental sites in distinct ecological regions will enable quantitative monitoring of key performance indicators, including release kinetics, microbial viability, rhizosphere colonization capacity, and sustained disease control efficacy throughout the crop growth cycle [125]. These field data are essential for validating laboratory findings and assessing the influence of real-world environmental variability on system performance.

The application of multi-omics and AI for mechanistic understanding and prediction is becoming increasingly recognized as a powerful approach. The complexity of field ecosystems demands more powerful analytical tools than conventional methods can provide. For example, metagenomic analysis can reveal how the gradual degradation of gel materials alters the composition and functional potential of indigenous soil microbial communities, providing direct evidence for ecological safety assessment [126]. Metabolomic profiling of encapsulated microbes can reveal key metabolic pathways affected by long-term entrapment, offering molecular-level insights into the mechanisms underlying loss of viability that can guide formulation optimization [127].

A full life-cycle assessment (LCA) should be conducted to quantify the environmental footprint of gel encapsulation systems, from raw material extraction and synthesis to field application and final degradation. LCA enables comparisons of environmental trade-offs between biopolymer-based formulations and conventional chemical pesticides, thereby supporting informed decision-making for sustainable agriculture [128, 129].

In summary, the convergence of long-term field trials, multi-omics analyses, AI-assisted modeling, and LCA will provide a multi-scale cognitive framework spanning from molecular mechanisms to ecosystem-level impacts. This integrated approach will advance the fundamental understanding of gel encapsulation systems. It will also accelerate their translation from laboratory concepts to practical, sustainable crop protection tools within the framework of smart agriculture [128, 130].

## Data Availability

Not applicable.
